# Medication adherence, self-care behaviour and knowledge on heart failure in urban South Africa: the Heart of Soweto study

**Published:** 2010-04

**Authors:** Ruf Verena, Simon Stewart, Sandra Pretorius, Maureen Kubheka, Karen Sliwa, Christine Lautenschläger, Peter Presek

**Affiliations:** Soweto Cardiovascular Research Unit, Department of Cardiology, Chris Hani-Baragwanath Hospital, Soweto, Johannesburg, South Africa; Institute of Pharmacology, Division of Clinical Pharmacology, Martin-Luther University Halle-Wittenberg, Halle (Saale), Germany; Soweto Cardiovascular Research Unit, Department of Cardiology, Chris Hani-Baragwanath Hospital, Soweto, Johannesburg, South Africa; Baker Heart Research Institute, Melbourne, Australia; Soweto Cardiovascular Research Unit, Department of Cardiology, Chris Hani-Baragwanath Hospital, Soweto, Johannesburg, South Africa; Soweto Cardiovascular Research Unit, Department of Cardiology, Chris Hani-Baragwanath Hospital, Soweto, Johannesburg, South Africa; Soweto Cardiovascular Research Unit, Department of Cardiology, Chris Hani-Baragwanath Hospital, Soweto, Johannesburg, South Africa; Institute for Medical Epidemiology, Biometrics and Computer Science, Martin-Luther University Halle-Wittenberg, Halle (Saale), Germany; Institute of Pharmacology, Division of Clinical Pharmacology, Martin-Luther University Halle-Wittenberg, Halle (Saale), Germany

**Keywords:** medication adherence, self-care behaviour, knowledge, heart failure, Africa

## Abstract

**Background:**

There is a paucity of data on treatment adherence in patients with chronic heart failure (CHF) in Africa.

**Methods:**

We examined the pattern of treatment adherence, self-care behaviour and treatment knowledge in 200 consecutive patients with CHF attending the Chris Hani Baragwanath Hospital, Soweto, South Africa via a combination of questionnaire (100%, *n* = 200) and pill count (41%, *n* = 82).

**Results:**

Mean age was 56 ± 14 years, 157 were black African (79%) and 109 (55%) were male. CHF-specific treatment included loop diuretics (93%), beta-blockers (84%), ACE inhibitors (74%), spironolactone (64%) and cardiac glycosides (24%); mean number of medications was 6 ± 2. Overall, 71% (58 of 82) adhered to their prescribed CHF regimen and individual medication adherence ranged from 64 to 79%. Behavioural adherence varied from 2.5 to 98%. Patient treatment knowledge was poor; 56% could not name medication effects or side effects. However, an average knowledge score of 69% was achieved on 10 questions concerning CHF management.

**Conclusion:**

As in other regions of the world, non-adherence to complex CHF treatment is a substantial problem in Soweto. Our data confirm the need for a dedicated CHF management programme to optimise CHF-related outcomes in a low-resource environment.

## Summary

Although the population burden and individual impact of chronic heart failure (CHF) has been well described in the western world,^1^ it has been less well described on the African continent.^2^,^3^ Significantly, CHF represents an emerging problem in low- to middle-income countries in sub-Saharan Africa undergoing epidemiological transition.^3^ For example, CHF is already an important cause of morbidity and mortality in black South Africans and it is conceivable that the incidence of CHF will increase over time.^4^ In addition to the need for a series of studies in Africa that parallel the detailed documentation of the epidemic of heart failure in the western world,^5^ we also need to better understand the individual experiences of those affected by heart failure in African communities. In this context, one of the key issues that determine individual outcomes is patient knowledge and adherence to prescribed gold-standard, non-pharmacological and pharmacological treatments.^6^

Results from high-income countries have shown that poor adherence to medical recommendations remains a substantial problem among people with CHF who must follow a multicomponent treatment regimen that includes medications, dietary restrictions and exercise recommendations.^7^ Overall, it has been estimated that between one-third and one-half of all patients with chronic heart conditions have difficulty adhering to their prescribed medication regimen in the western world, contributing to impaired quality of life, high healthcare costs linked to increasing rates of hospital re-admissions and out-patient hospital care, in addition to premature mortality.^8^ Importantly, increased CHF-related knowledge is associated with better treatment adherence.^9^ However, despite its potential clinical importance (there is no reason to suspect African patients are immune to this problem), there is a paucity of data on treatment adherence in patients with heart disease in the African context.^10^-^12^

## Methods

It is within the above context that we examined patterns of adherence to prescribed pharmacological and non-pharmacological therapy in a large cohort of black African patients in Soweto, diagnosed with CHF. We also examined their understanding of prescribed treatment and the overall purpose of CHF management.

This represents a key substudy of the previously described Heart of Soweto study,^3^,^13^ which is currently mapping the emergence and spectrum of heart disease in a black urban population. This study benefits from the unique setting at the Chris Hani-Baragwanath Hospital, representing the only tertiary-care centre for the population of Soweto and surrounding communities.

Soweto, a township situated in the south west of Johannesburg, South Africa, has a population of 1.1 million, which has undergone economic development, enabling a large proportion of its population to achieve a more affluent lifestyle. The township comprises people from different ethnic backgrounds, however the black African population is predominant.

As Chris Hani-Baragwanath Hospital is the only point of specialist cardiac care for the population of Soweto, the cardiology clinic probably attends to nearly every patient presenting with symptoms evolving from a cardiac condition. The population of the cardiology out-patient department consists of patients with a suspected cardiac disorder, seen and referred by 12 local Soweto primary-care clinics. Other patients are initially seen at the general medicine out-patient facilities, specialist medical registrar clinic, diabetes clinic or are in-patients admitted to any other ward of the Chris Hani-Baragwanath hospital and need a cardiac consultation.

During November 2006 and April 2007, we recruited 200 consecutive patients with a confirmed diagnosis of CHF presenting at the cardiology clinic. For the purpose of this study, patients were included if they had a confirmed diagnosis of CHF by an attending cardiologist, based on typical clinical symptoms (shortness of breath, oedema, fatigue) and a documented left ventricular ejection fraction (LVEF) of ≤ 45% using echocardiography. Non-English-speaking patients were excluded only if a translator was unavailable. Patients were approached in the out-patient cardiology clinic and invited to participate. Data were collected after an informed consent form was signed.

Prior to study commencement, the relevant local Ethics of Human Research Committee approved the study. The study conforms to the standard statements on ethics outlined by the Declaration of Helsinki.

During a pilot study consisting of 20 participants, a ‘medication adherence and knowledge on heart failure’ survey specific to this predominantly black African community was developed in October 2006. The questionnaire was then applied to the above study population on a one-to-one basis in an interview of approximately 20 minutes. All interviews were executed by the same investigator (VR) and if needed, a translator assisted the applicant.

A pill count of prescribed CHF treatment was conducted in those 82 patients (41%) who returned for a scheduled one-month post-interview appointment. If appropriate, a telephone reminder was made two days prior to this appointment; however, contact details were available for few patients.

The medication adherence and knowledge on heart failure survey addresses the following sections: demographic and clinical data, medication adherence, self-care behaviour (adherence to follow-up appointments, weighing behaviour, dietary restriction, regular physical activity, smoking abstinence and alcohol intake), knowledge concerning CHF medication and overall CHF management. Questionnaires in other studies included the same sections,^7^,^9^,^14^ however the answer possibilities in our questionnaire were mostly dichotomous (yes or no) instead of offering a range of answers (no, a little, some, a lot).

In accordance with previous studies of this type,^7^,^14^ we defined treatment adherence as ≥ 75% of the prescribed pills taken. Similarly, we defined appointment adherence as being present at ≥ 75% of the assigned appointments consisting of quarterly check-ups and monthly medication refills at the hospital pharmacy. In accordance with the European Society of Cardiology guidelines,^6^ we defined behavioural adherence as daily weight monitoring, daily intake of five servings of fruit and vegetables, drinking less than two litres of fluids per day, being physically active, with compensated CHF two to three times per week, refraining from smoking and keeping a moderate alcohol intake (one beer, one to two glasses of wine per day).

## Statistics

Descriptive statistics and measures of frequency were conducted in Microsoft EXCEL® and were used to describe the study population and various adherences. Data are presented as means ± standard deviation or percentages. To compare groups we used χ^2^ analyses for discrete variables and the Student’s *t*-test for continuous variables. Binary logistic regression models were performed in SPSS 11.5 to determine variables predicting adherence. Determinants for medication adherence were presented by odds ratio (OR) and 95% confidence intervals (CI), where an OR = 1 indicated no influence on medication adherence. Significance was accepted at the two-sided level of 0.05.

## Results

The overall demographic and clinical profile of the study cohort is presented in Table 1. Overall, black Africans predominated [*n* = 157 (79%)] and there were more men [*n* = 109 (55%)] than women [*n* = 91 (45%)] with no difference in age profile (mean age 56 ± 13 vs 56 ± 15 years). Apart from black African patients there were Asian Indians (*n* = 10), coloureds (*n* = 8) and white Africans (*n* = 25), which we combined as ‘other races’. Almost half of the patients were retired and nearly all lived in a shared household. Black Africans were significantly more likely to have no or standard education than the other races combined [128 (82%) vs 24 (56%), *p* = 0.001].

**Table 1.** Sociodemographic And Clinical Profile

	*Total (%) (n = 200)*	*Men (%) (n = 109)*	*Women (%) (n = 91)*	*Black African (%) (n = 157)*	*Other races (%) (n = 43)*
Education profile					
None	16 (8.0)	9 (8.3)	7 (7.7)	14 (8.9)	2 (4.6)
Standard 1–5	43 (22)	18 (17)	25 (28)	42 (27)	1 (2.3)
Standard 6–10	93 (47)	49 (45)	44 (48)	72 (46)	21 (49)
Matriculation/post matriculation	42 (21)	30 (28)	12 (13)	25 (16)	17 (40)
Employment status					
Employed	54 (27)	37 (34)	17 (19)	39 (25)	15 (35)
Unemployed	57 (29)	30 (27)	27 (30)	49 (31)	8 (19)
Retired	89 (45)	42 (39)	47 (51)	69 (44)	20 (47)
Living environment					
Alone	18 (9.0)	12 (11)	6 (6.6)	13 (8.3)	5 (12)
Perceived practical support					
Not at all	36 (18)	21 (19)	15 (17)	28 (18)	8 (19)
A little or some	36 (18)	17 (16)	19 (21)	34 (22)	2 (4.6)
A lot	124 (62)	68 (62)	56 (62)	91 (58)	33 (77)
Perceived emotional support					
Not at all	16 (8.0)	14 (13)	2 (2.2)	12 (7.6)	4 (9.3)
A little or some	41 (21)	23 (21)	18 (20)	32 (20)	9 (21)
A lot	137 (69)	66 (61)	71 (78)	108 (69)	29 (67)
Clinical profile					
Mean LVEF (%) ± SD	32 ± 8	32 ± 8	33 ± 8	32 ± 8	34 ± 7
NYHA class II/III	180 (90)	97 (89)	83 (91)	141 (90)	39 (91)
NYHA class IV	5 (2.5)	2 (1.8)	3 (3.3)	4 (2.5)	1 (2.3)
Newly diagnosed HF	60 (30)	32 (29)	28 (31)	21 (13)	6 (14)
Treated for HF > 1 year	140 (70)	77 (71)	63 (69)	136 (87)	37 (86)
Prior admission for HF	169 (85)	92 (86)	77 (85)	135 (86)	34 (79)
Prescribed treatment					
Beta-blocker	168 (84)	93 (85)	75 (82)	129 (82)	39 (91)
ACE inhibitor	148 (74)	79 (72)	69 (76)	117 (75)	31 (72)
Loop diuretic	185 (93)	97 (89)	88 (97)	150 (96)	35 (81)
Spironolactone	127 (64)	68 (62)	59 (65)	103 (66)	24 (56)
Cardiac glycoside	47 (24)	25 (23)	22 (24)	41 (26)	6 (14)

When questioned about their self-perceived level of social support, black Africans were less likely to report having ‘a lot of’ practical support than other races combined [91 (58%) vs 33 (78%), *p* = 0.038], however there was no major difference found in respect of reported emotional support.

## Clinical profile

Overall, 90% of our study patients were classified as New York Heart Association functional class (NYHA) II and III at the point of being diagnosed with CHF. Overall, the mean left ventricular ejection fraction was 32 ± 8%. Black Africans were less likely to live longer than five years with CHF than the other races combined [61 (39%) vs 23 (53%), *p* = 0.085] and additionally, they were more likely to have been admitted to hospital before the point of investigation due to their CHF [135 (86%) vs 34 (79%), *p* = 0.188]. However, that did not reach statistical significance.

**Fig. 1.**
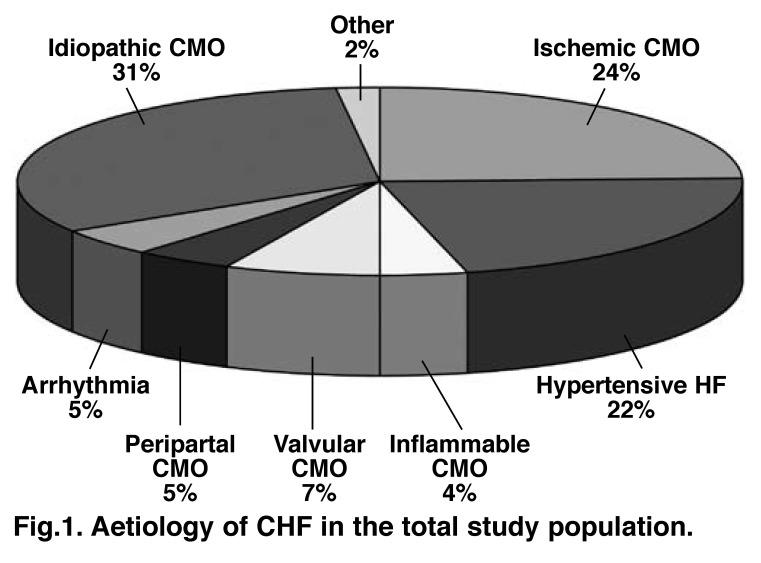
Aetiology of CHF in the total study population.

As represented in Fig. 1, the three most common underlying aetiologies for CHF in our study population were idiopathic cardiomyopathy (CMO), ischaemic CMO and hypertensive heart failure. Of the 24% diagnosed with ischaemic CMO, 45% were black African and 55% were other races combined (thereof 12% Asian Indians, 10% coloureds and 33% white Africans). Other causes of CHF included post partum CMO (5% of patients), a condition more commonly found in Africa.^2^

## Treatment adherence

Standard CHF treatment included beta-blockers (84%), ACE inhibitors (74%), loop diuretics (93%), spironolactone (64%) and cardiac glycosides (24%). Other medications commonly prescribed in patients with CHF included potassium supplements (54%), aspirin (47%), lipid-lowering agents (33%), warfarin (19%), hypoglycaemic agents, thiamine supplements and calcium antagonists (14%), and the anti-arrhythmic agent amiodarone (7%). Study participants were prescribed a mean of 6 ± 2 individual medications.

**Fig. 2.**
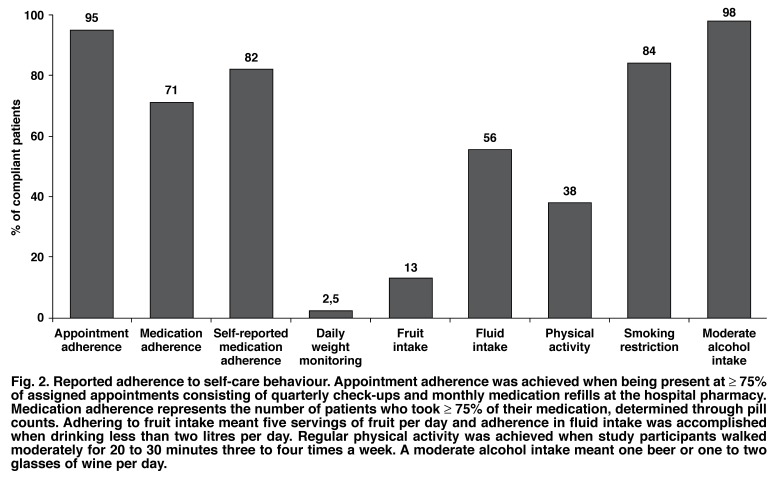
Reported adherence to self-care behaviour. Appointment adherence was achieved when being present at ≥ 75% of assigned appointments consisting of quarterly check-ups and monthly medication refills at the hospital pharmacy. Medication adherence represents the number of patients who took ≥ 75% of their medication, determined through pill counts. Adhering to fruit intake meant five servings of fruit per day and adherence in fluid intake was accomplished when drinking less than two litres per day. Regular physical activity was achieved when study participants walked moderately for 20 to 30 minutes three to four times a week. A moderate alcohol intake meant one beer or one to two glasses of wine per day.

Overall, 82% of the study participants reported that they were compliant with their prescribed medication (Fig. 2) and 16% acknowledged not taking ≥ 75% of their prescribed CHF treatment. There was a difference between men and women with 85 versus 75% of participants, respectively, rating themselves as medication compliant.

Although adherence to follow-up appointments given by the attending cardiologist was good (Fig. 2), there was a poor adherence to the appointment given for the pill count with only 82 of 200 patients returning (41%). These pill counts revealed that 71% were clearly compliant and 22% non-compliant with their overall prescribed HF regimen (a pill count was not possible in 7% of this subgroup of participants).

**Fig. 3.**
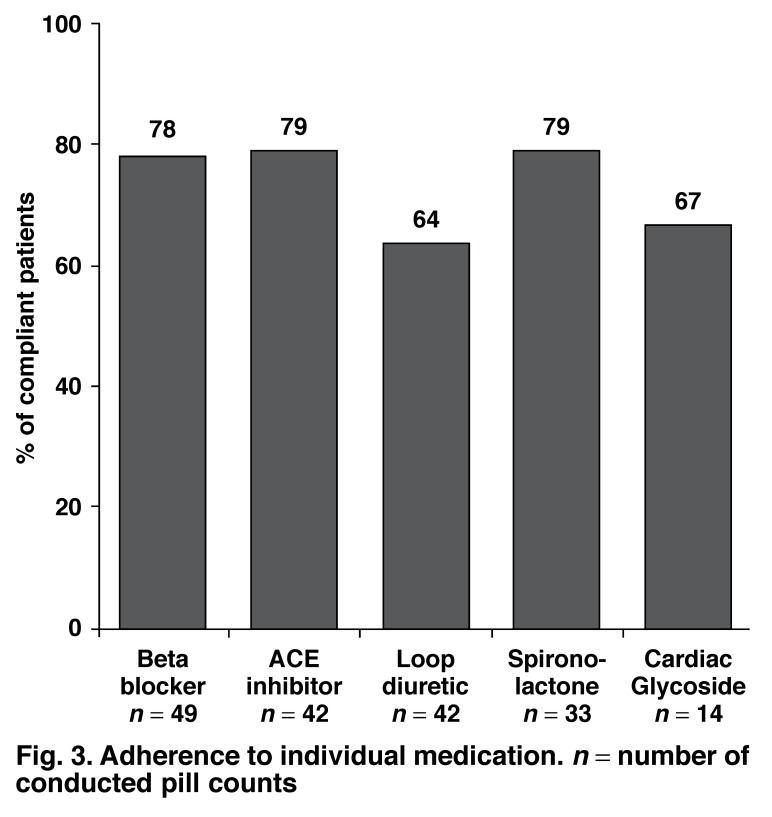
Adherence to individual medication. n = number of conducted pill counts

Fig. 3 shows that the highest adherence rates were for ACE inhibitors and spironolactone compared to the lowest for loop diuretics. Participants reported skipping their loop diuretic (furosemide) most often, with forgetfulness and avoidance of side effects being the most common reasons for non-adherence overall.

## Determinants for treatment adherence

There were no statistically significant factors linked to treatment non-adherence on the basis of multiple logistic regression analysis. However, those participants reporting less than three of the symptoms commonly found in patients with CHF were 4.5-fold (95% CI: 0.95–21.7; *p* = 0.058) more likely to be designated as compliant, demonstrating the importance of treatment for symptom control and likely clinical benefit of prescribed treatment. At the same time, men tended to be more compliant than women (OR 1.8, 95% CI: 0.61–5.14; *p* = 0.294) without approaching significance.

## Self-care behavioural adherence

Fig. 2 demonstrates adherence to different self-care behaviours. Overall, 95% of all study participants kept ≥ 75% of their follow-up appointment schedule and were consequently designated as compliant. Overall, 81% adhered to 100% of their follow-up appointment schedule. There was no difference found between men and women and no major difference between the races in this regard.

Daily weight monitoring was associated with the lowest rate of adherence (only 19% of participants having a scale at home), while avoiding alcohol intake was associated with the highest rate of adherence. From a dietary behaviour perspective, 87% of participating patients were non-compliant concerning daily intake of fruit. However, 64% of participants reported having difficulty affording fresh fruit. From a smoking perspective, there were 31 (16%) current smokers and 59 (30%) former smokers.

## Health education

**Fig. 4.**
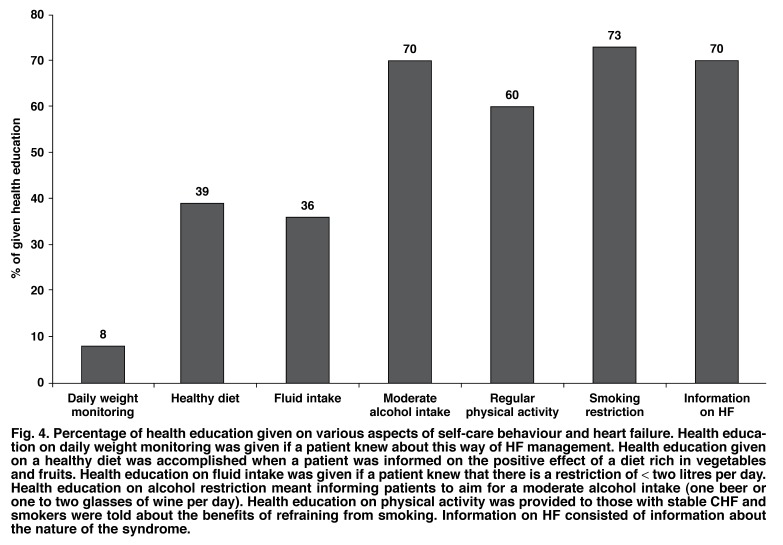
Percentage of health education given on various aspects of self-care behaviour and heart failure. Health education on daily weight monitoring was given if a patient knew about this way of HF management. Health education give on a healthy diet was accomplished when a patient was informed on the positive effect of a diet rich in vegetables and fruits. Health education on fluid intake was given if a patient knew that there is a restriction of < two litres per day. Health education on alcohol restriction meant informing patients to aim for a moderate alcohol intake (one beer or one to two glasses of wine per day). Health education on physical activity was provided to those with stable CHF and smokers were told about the benefits of refraining from smoking. Information on HF consisted of information about the nature of the syndrome.

The medication adherence and knowledge on heart failure survey contained questions in several sections on health education provided by a doctor or a nurse. According to the patient’s responses, the least amount of education was provided in relation to daily weight monitoring, with only 8% of participants remembering this component of education. Dietary management was also less memorable than education focusing on alcohol, smoking and CHF in general (Fig. 4).

## Knowledge on heart failure medication and management

Overall, patient knowledge concerning their prescribed CHF medication was poor: 56% could not name the effect or any side effects of their medication. On average, only 10% of effects and side effects of pills taken could be named by all participating patients. However, in those patients who self-reported receiving health education on CHF (70%, Fig. 4), the average knowledge on CHF medication was twice as high as in patients without health education. An average score of 69% was achieved on 10 questions concerning CHF management. Percentages on correct answers for each of the 10 questions varied between 29 and 89%. Again, patients who received education on CHF management had a higher average knowledge (70%) than patients who had not received information on CHF management (65%).

## Discussion

To our knowledge, this represents the largest report to date on the pattern of treatment adherence and knowledge in predominantly black African patients with CHF emanating from South Africa. Indeed, it is one of the largest overall reports relating to CHF from the continent.^11^ This is particularly significant given our recent reports of a higher-than-expected burden of CHF in the urban South African community of Soweto,^13^ and the likelihood of an increase in both traditional and affluent forms of the syndrome due to epidemiological transition.^13^

How do our data compare with data derived from western cohorts? Our findings on medication adherence are generally lower than reports emanating from high-income countries.^8^ However, a study conducted in Zimbabwe attained results that supported our findings:^11^ Bhagat and Mazayi-Mupanemunda found that 73% of the 22 investigated heart failure patients were considered compliant with their prescribed medication.^11^ Although adherence rates from several studies in the western world vary from 71 to 99%, over half of the listed studies had an adherence rate above 80%.^14^-^20^ Alternatively, studies from Sweden (71%) and the USA (73%) have reported similar rates to the current study.^15^,^20^

When looking at individual medication adherence to the five basic medications prescribed for CHF, our results ranged from 64 to 79%. In a UK study, Struthers *et al*. found that 18, 34 and 58% of patients had adherence rates of < 70%, < 85% and 100%, respectively, to prescribed ACE inhibitors.^21^ In contrast, Monane *et al*. found that only 10% of their study patients were fully compliant with prescribed digoxin therapy during one year of follow-up.^22^

With regard to adherence to appointment schedules, patients in this study were similar to most other reports (i.e. > 90% appointment adherence).^7^,^14^ Alternatively, our data on daily weight monitoring were generally lower than that of other reports (ranging from 12 to 75%).^23^,^24^ The fact that only 19% of our study patients had a scale at home is an important factor in this regard. In respect of fluid management, 56% of our study patients reported adhering to the recommended fluid intake of less than two litres per day. In comparison, Artinian *et al.* (23%)^18^ and Jaarsma *et al.* (37%)^19^ reported lower, while van der Wal (73%)^25^ reported higher adherence rates to fluid restriction.

With 38% of participating patients performing some form of regular exercise (especially walking 20 to 30 min per day), our results were lower than results from other studies (equivalent range 39 to 67%).^7^,^8^,^14^,^19^,^26^ Concerning smoking abstinence, 16% of our participating patients persisted in smoking tobacco. In comparison Evangelista *et al.* and Carlson *et al.* found less than 10% of their study patients to be non-compliant in this regard,^7^,^14^,^26^ whereas higher smoking rates have been reported by Artinian *et al.* (46%) and also by Evangelista *et al.* (55%) in a study on veterans with CHF.^18^,^27^

Overall, 98% of all study participants were compliant with regard to reduced alcohol intake. This result is higher than in studies led by Evangelista *et al.* where adherence to alcohol limitation varied between 64 and 94%,^7^,^14^,^27^ compared to 56% in a cohort studied by Artinian *et al.*^18^

Our finding that men tended to be more compliant with prescribed medication is both supported^15^,^28^ and refuted^22^,^29^ by other studies, with no clear pattern in the literature. Although it is logical to suggest that those with greater symptoms are more likely to be non-compliant, this association is not a regular feature in the literature.

It is now largely accepted that greater CHF-related knowledge has a positive impact on adherence behaviours.^9^,^25^ Unfortunately, our data imply that patient education at the cardiology out-patient department of the Chris Hani-Baragwanath Hospital is suboptimal in respect of a number of key educational areas. A similar result was found in another study from South Africa focusing on patients with hypertension.^12^ There were some encouraging results with regard to the provision of CHF information, but this may be due to the fact that study participants had to decide only whether a statement on CHF management was correct or incorrect.

In a study lead by Ni *et al.*, the percentage of patients choosing the correct answer on eight questions concerning CHF management varied between 43 and 90% versus a range of 29 to 89% in our study.^9^ By self-report, 68% of our patients said they knew a little or nothing about CHF. Ni *et al.* found that only 38% of their study participants reported that they knew only a little or nothing about CHF.^9^

It is clear from this and other studies from the western world that poor adherence to treatment and CHF-related self-care behaviour exposes the patient to an increased risk of clinical instability and increased symptoms.^16^,^30^ This can result in higher-than-expected hospital admission rates, which place a substantial (cost) burden on the healthcare system.^16^,^31^ In order to prevent the deterioration of the patient’s condition, adherence to medication and other self-care behaviour needs to be ameliorated.

In the western world, CHF management programmes have had a positive effect on outcomes of adherence, using various strategies.^19^,^32^,^33^ These data certainly support the potential for CHF management programmes to improve health behaviours and outcomes in South Africa. We plan to use these data to undertake one of the first randomised, controlled trials of CHF management in Africa with interventions suited to the local culture and environment.

## Limitations

There are a number of study limitations that require comment. Although, pill counts are a fairly objective method to measure medication adherence, it is still prone to errors such as the assumption that a pill was truly taken if it is not in the medication box. Serum bioassays or electric monitoring advices may be more objective and thus more accurate.^14^ However, in our settings, pill counts and interviews were the only options for measuring adherence.

As we also based our adherence figures on the results from the pill counts, the findings may be biased, given that those patients who returned for the pill count were already more likely to be compliant in following instructions and were therefore more prone to follow advice concerning medication adherence. Similarly, as the interview took place before the pill count, patients might have paid more attention to taking their medication regularly during the following month. As a result, medication adherence may be overestimated in our study. In future studies, pill counts could be conducted at one, three and six months after the interview to obtain more accurate results.

As self-reporting is always subjective and biased, adherence rates to self-care behaviour and the measured knowledge on CHF and its management may have been affected. We also defined behavioural adherence in accordance with the European Society of Cardiology guidelines, even though they might not be applicable to our study population, considering the different disease profiles, cultural and socio-economic profiles and financial backgrounds.

Another limitation to our study was the diversity of languages in South Africa (11 official languages). A translator was used in various interviews.

Finally, it is difficult to ascertain how representative these data are in relation to other African centres. For example, there was a relatively large number of patients with ischaemic CMO, a generally uncommon cause of CHF in Africa. However, we have recently reported a rise in such cases,^34^ and the other common causes of CHF in Africa (idiopathic CMO and hypertensive heart failure) were well represented. Given that this was a relatively small group of patients (although large for Africa), we were most probably underpowered to fully explore predictors of non-adherence.

Despite these limitations, our study is the first study of this dimension on medication adherence in South Africa. These data indicate the need for interventions that have already been established in the western world to improve health outcomes. These data therefore support the need for culturally sensitive and affordable CHF management programmes that can improve treatment adherence and optimise self-care behaviours and knowledge, in order to improve CHF-related health outcomes overall in South Africa.

## Conclusion

As in many other regions of the world, non-adherence to complex CHF treatment is a substantial problem in Soweto. Additionally, as education on heart failure is not optimal, knowledge on heart failure medication and management is poor. These data confirm the need for a dedicated heart-failure programme in a lowresource set up to optimise CHF management and outcomes.
